# Feasibility of a pilot preceptorship programme for pharmacists working towards the advanced career stage in Scotland: a mixed-methods study

**DOI:** 10.1007/s11096-026-02222-z

**Published:** 2026-09-18

**Authors:** Mairi-Anne McLean, Paul Forsyth, Chris F. Johnson, Anne C. Boyter

**Affiliations:** 1 University of Strathclyde, Glasgow, UK; 2 NHS Greater Glasgow and Clyde, Glasgow, UK; 3 Golden Jubilee National Hospital, Clydebank, UK

**Keywords:** Advanced practice, COM-B model for behavioural change, Pharmacy, Preceptorship

## Abstract

**Introduction:**

Structured preceptorship programmes may support pharmacists working towards the advanced career stage, yet evidence on feasibility remains limited.

**Aim:**

The aim of this study was to evaluate the feasibility of a pilot preceptorship programme for pharmacists working towards the advanced career stage.

**Method:**

An embedded mixed-methods pilot study was conducted in primary care practices within one territorial health board in NHS Scotland, recruiting four preceptor–preceptee pairs for a 16-week preceptorship programme. Quantitative data assessed programme delivery, including joint clinical sessions, shadowing sessions and changes in preceptee-reported self-efficacy and competence from baseline to 16 weeks. Qualitative data from semi-structured interviews with preceptors, preceptees, and senior pharmacy management explored perceptions of implementation barriers and enablers. Data were analysed using the COM-B framework, which considers Capability, Opportunity, and Motivation as key influences on behaviour.

**Results:**

Feasibility assessment demonstrated incomplete implementation, with preceptee-reported delivery of 14 of 60 anticipated joint clinical sessions (including both preceptee led clinics and shadowing sessions) and 3 of 12 anticipated structured review meetings over the 16-week programme. Self-reported self-efficacy and competence data were collected over the study period; however, due to incomplete implementation, these data are not interpreted as evidence of programme effect. Qualitative analysis identified participants’ perceptions of barriers to implementation, including competing workload priorities, lack of protected time, logistical challenges, limited stakeholder engagement, and insufficient holistic feedback (as feedback primarily focused on clinical rather than practical aspects), alongside enablers such as access to resources and the perceived value of preceptorship.

**Conclusion:**

The pilot preceptorship programme was not fully feasible in this professional context, highlighting important considerations for future implementation. Despite incomplete delivery, participants reported perceived changes in self-reported self-efficacy and competence; however, these findings do not provide evidence of programme effect, highlighting the need for further refinement and evaluation of preceptorship models in advanced pharmacist practice.

**Supplementary Information:**

The online version contains supplementary material available at https://doi.org/10.1007/s11096-026-02222-z.

## Impact statements

- This pilot preceptorship programme highlights the importance of assessing feasibility when implementing advanced practice preceptorship programmes.
- COM-B analysis identified perceived implementation barriers, including workload pressures, lack of protected time, limited stakeholder engagement, and challenges integrating preceptorship into current pharmacist roles.

This pilot informs future evaluation and quality improvement by identifying areas to refine preceptorship programmes in advanced pharmacist practice.

## Introduction

Pharmacists’ roles continue to expand internationally, with increasing expectations to deliver advanced, holistic, autonomous care for people with complex therapeutic needs, reflecting broader shifts towards advanced, patient-centred practice [1–5]. In this paper, an “advanced pharmacist” is defined as a pharmacist accredited against an advanced practice framework [6]. Such pharmacists possess the additional knowledge and skills required to deliver holistic, patient-centred care to individuals and/or groups with highly complex needs, while autonomously managing risk in situations of significant uncertainty where evidence may be limited or ambiguous [6].

Internationally, health workforce development strategies, including the World Health Organization Global Strategy on Human Resources for Health: Workforce 2030 [3], emphasise strengthening workforce capacity, education, and competency development to support evolving healthcare needs. In pharmacy specifically, the International Pharmaceutical Federation Global Advanced Development Framework provides an internationally recognised framework to support progression towards advanced practice and workforce development [7].

In the UK, pharmacists are increasingly expected to complete advanced practice frameworks such as the Royal Pharmaceutical Society (RPS) Core Advanced Curriculum, which defines generic advanced-level capabilities and provides a route for formal credentialling [6]. The curriculum emphasises autonomous care for patients with complex needs and additional elements of risk or clinical uncertainty [6]. Although independent prescribing is not explicitly mandated, it is treated as an integral component of advanced practice [6, 8]. As pharmacists’ roles expand across medicines optimisation, long-term condition management, acute presentations, and complex polypharmacy, there is increasing recognition that structured support is needed to ensure confident progression towards advanced practice [9–12].

Despite expectations for pharmacists to demonstrate advanced-level capabilities, support for their development appears to vary across settings [12]. High workloads, limited protected time, unstructured supervision, and variable delegation of clinical duties may impede progression [13, 14], while evidence on how to support pharmacists undertaking advanced practice programmes remains limited [9, 12]. Together, these factors highlight the need for structured clinical supervision to support confidence, capability, and organisational assurance.

Preceptorship programmes, structured, time-bound programmes designed to support capability growth, reflection, and role development, have been widely used in nursing and early-career pharmacy [13–15]. They are associated with improvements in confidence, competence, and integration into clinical teams [15–19]. However, limited evidence exists regarding application for pharmacists working towards advanced roles, where developmental needs may be more complex and context dependent.

Recent research in Scotland used a three-phase development process involving a literature review, expert consensus, and a two-round modified Delphi survey of the Scottish pharmacy workforce to generate a consensus-derived set of features of an advanced-level pharmacist preceptorship programme, from which the pilot programme evaluated in this study was developed [18]. The programme incorporated established preceptorship principles of supervised clinical practice, direct observation, preceptor feedback, and structured review meetings to support progression towards autonomous advanced practice. However, as pharmacists working towards advanced roles continue to report organisational and contextual challenges, including limited protected time, competing service demands, and variable support, the current work environment and culture may impede the development of practice [12, 13, 20, 21].

### Aim

The aim of this study was to evaluate the feasibility of a pilot preceptorship programme for pharmacists working towards the advanced career stage.

## Method

Study setting: The study was conducted within a territorial health board in NHS Scotland, part of the publicly funded UK National Health Service, with participants based in primary care general medical practices.

An embedded mixed methods approach was used to evaluate the pilot [22]. Consistent with feasibility study guidance, the study focused on whether the preceptorship programme could be delivered as intended within routine practice settings and on identifying factors influencing implementation [23, 24]. The quantitative phase aimed to assess programme feasibility through measurement of delivery against expected activity and also recorded changes in self-reported self-efficacy and competence. The qualitative phase aimed to explore participants’ experiences of programme implementation and identify factors influencing implementation.

### Sampling and recruitment

Stage 1 involved pharmacists nominated to take part in the pilot by senior managers in the participating health board. Senior managers were asked to nominate pharmacists who met the preceptor and preceptee inclusion criteria (Tables 1, 2), which were derived from a published modified Delphi study [18] that provides a foundation for the development of preceptorship programmes for pharmacists working towards the advanced career stage. The number of eligible pharmacists was not predefined. Those who consented were screened against inclusion criteria. Stage 2 invited all six participants who completed Stage 1, and two senior managers overseeing the programme (n = 8), to participate in qualitative interviews.

**Table 1:** Preceptee inclusion criteria

Preceptee	Delphi—Consensus met	Delphi –Consensus not met	Not directly assessed in Delphi; derived from programme development intent
Is a registered pharmacist			✓
Is in a patient facing role			✓
Is an independent prescriber		✓	
Working towards advanced career stage			✓
Has an open RPS Core Advanced e-portfolio			✓
Demonstrates self-motivation	✓		
Demonstrates active participation	✓		
Demonstrates evidence of person-centered practice	✓		
Demonstrates the ability to relate current experience to previous experience	✓		
Demonstrates leadership skills		✓	
Demonstrates initiative	✓		
Demonstrates maturity	✓		
Demonstrates professionalism	✓		
Demonstrates good judgement	✓

**Table 2:** Preceptor inclusion criteria

Preceptor	Delphi—Consensus met	Delphi—Consensus not met	Not directly assessed in Delphi; derived from programme development intent
Is a registered pharmacist		✓	
Is in a patient facing role			✓
Has relevant experience at advanced practice level	✓		
Has competence at the advanced practice level or above in a relevant clinical area	✓		
Has confidence in the delivery of advanced practice	✓		
Has relevant experience of teaching clinical practice	✓		
Currently delivers clinical practice at the advanced practice level or above	✓		
Shows willingness to undertake the preceptor role	✓		
Has characteristics of a role model	✓		
Can demonstrate evidence of person-centered practice	✓		
Has excellent interpersonal skills	✓		

Participation in either stage was voluntary, and all individuals invited received study information and provided consent.

### Intervention

The programme was informed by a published three-phase preceptorship programme development study which involved literature review, expert consensus, and two-round modified Delphi survey [18].

Stage 1 was a 16-week programme comprising regular joint clinical sessions, three review meetings, practice development objectives, and evidence of progression against RPS Core Advanced Curriculum outcomes:

- 2.1—Deliver care using advanced pharmaceutical knowledge and skills
- 2.2—Undertake a holistic clinical review of individuals with complex needs, and
- 2.3—Demonstrate effective clinical reasoning skills, making autonomous, evidence informed, person-centred decisions.

The lead researcher met with participants to introduce them to the programme, clarify expectations, and answer questions. Participants were informed that joint patient-facing clinical sessions would typically occur weekly, although the timing and frequency could be adjusted to suit individual schedules. While no mandatory frequency was imposed, pairs were expected to complete approximately 15 joint sessions over the programme. Sessions were intended to be primarily preceptee-led, with initial shadowing if required. Pairs were expected to complete three review meetings (initial, interim, and final) to monitor progress.

### Data collection and analysis

Quantitative data were collected at baseline and every four weeks using Microsoft® Forms. Preceptee self-reported self-efficacy and competence were measured using 5-point Likert scales based on a validated General Self-Efficacy Scale hosted by Imperial College London [25] and the self-perceived competence scale described by Lai et al. [26]. Both instruments underwent minor contextual modifications to align with advanced pharmacist practice (see Supplementary Files). A Microsoft® Forms Activity Form captured clinic and review meeting activity, enabling longitudinal tracking across the pilot. Given the small sample size, data were analysed descriptively, with results reported as ranges.

*Stage 2*: Semi‑structured one‑to‑one interviews were conducted on Microsoft® Teams using a COM‑B-informed topic guide (see Supplementary Files). The topic guide was developed using the COM-B framework as the structure, with prompts to explore factors influencing implementation of the pilot programme. Transcripts were verified for accuracy.

Transcripts were analysed using a hybrid deductive–inductive approach informed by the COM-B framework. The COM-B domains of Capability, Opportunity, and Motivation were used as the deductive framework. Inductive coding identified codes which represented the lowest level meaning units and were grouped into themes. The coding framework and codebook are provided in the Supplementary Files.

Codes were classified as enablers when participants generally described them as present, barriers when they were described as absent, and neutral when views were mixed. This classification was undertaken separately for preceptees, preceptors, and other stakeholders. Themes were classified according to the predominant classification of their codes and mapped to COM-B domains to support interpretation.

Trustworthiness was supported through double coding (CJ) of an initial transcript, which informed refinement of the coding framework through discussion. Independent review of the final coding (CJ) and theme structure, alongside discussion of the methodological approach with supervisors (AB, PF), supported credibility, dependability and confirmability of the analytical process [27]. Saturation was not assessed due to the fixed, small sample.

All data were stored securely on the University of Strathclyde network.

Integration occurred at the interpretation stage of the study, where quantitative and qualitative findings were considered together to provide a more comprehensive understanding of programme implementation.

### Ethics approval

Ethics review was conducted by the Strathclyde University Institute of Pharmacy and Biomedical Sciences Ethics Committee. Ethical approval was granted for Stage 1 on 29 October 2024 and for Stage 2 on 13 May 2025. NHS Research Ethics Committee approval was not required.

## Results

Stage 1: The sample comprised four preceptor-preceptee pairs. One preceptee withdrew early leaving three pairs completing the intervention. Detailed demographic information is not reported due to risk of deductive disclosure given the small sample size.

Feasibility assessment demonstrated incomplete implementation. Fourteen of the predicted 60 planned joint clinical sessions (23%) were reported. Clinical delivery sessions (preceptee-led) under preceptor supervision were limited, with 1 session completed by two pairs and 4 sessions completed by another. Shadowing sessions, where preceptees observed preceptor-led clinics, were more frequent, with 2–4 sessions recorded per pair.

Three out of 12 predicted review meetings (25%) were reported, with one meeting conducted per completing pair.

Self-reported confidence and competence scores were collected across the study period; however, due to incomplete implementation, these data are not interpreted as evidence of programme effect. Scores were measured using 5-point Likert scales (see Supplementary Files). Changes in confidence scores ranged from 0 to + 3 points across the three outcomes. Changes in competence scores ranged from 0 to + 1 points across the three outcomes.

*Stage 2*: Participants represented a variety of roles including three preceptors, three preceptees and two senior managers. Detailed demographic information is not reported due to risk of deductive disclosure given the small sample size. Interviews lasted between 16 and 45 min.

Analysis using the COM-B framework identified a range of perceived barriers and enablers across the domains of Capability, Opportunity, and Motivation.

While the analysis focused primarily on preceptors and preceptees, a heterogeneous group of “other stakeholders” was identified (e.g. senior and national pharmacy leaders, the wider pharmacy profession and practice-based colleagues).

Qualitative analysis identified perceived barriers to implementation, including competing workload priorities, limited protected time and variable engagement, alongside enablers such as access to resources and the perceived value of preceptorship.

**Fig. 1:**
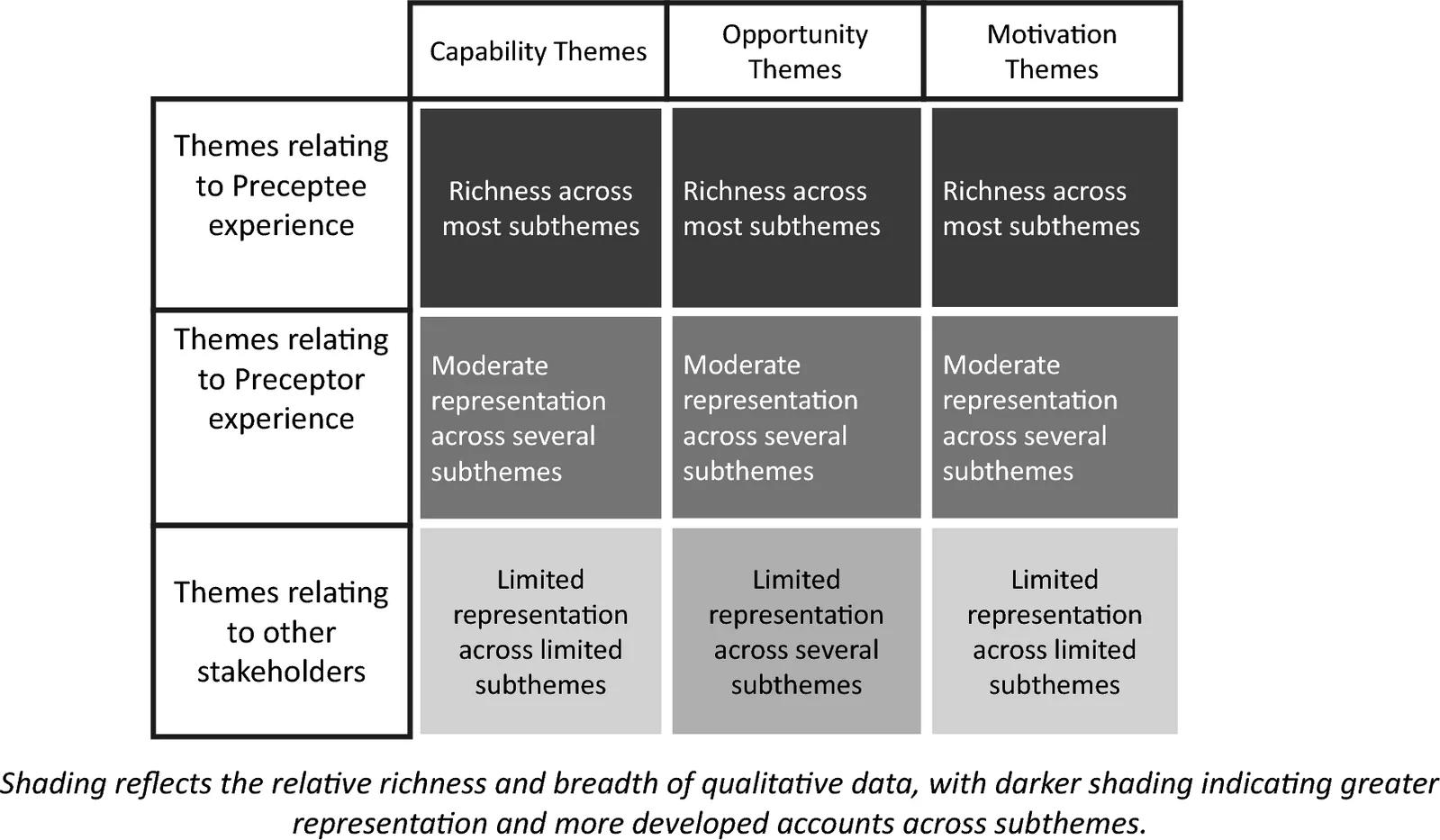
Relative richness and breadth of qualitative data across Com-B domains by stakeholder perspective

As shown in Fig. 1, qualitative data varied in richness and breadth of themes across factors relating to preceptees, preceptors and other stakeholders. Data relating to preceptees demonstrated greater representation and more developed accounts across a wider range of themes, whereas data relating to preceptors were less extensive and data relating to other stakeholders were comparatively limited. There is variation in the number of supporting quotations presented across the various perspectives below. Presenting this variation enhances trustworthiness by describing the richness of data relating to each grouping.

**Fig. 2:**
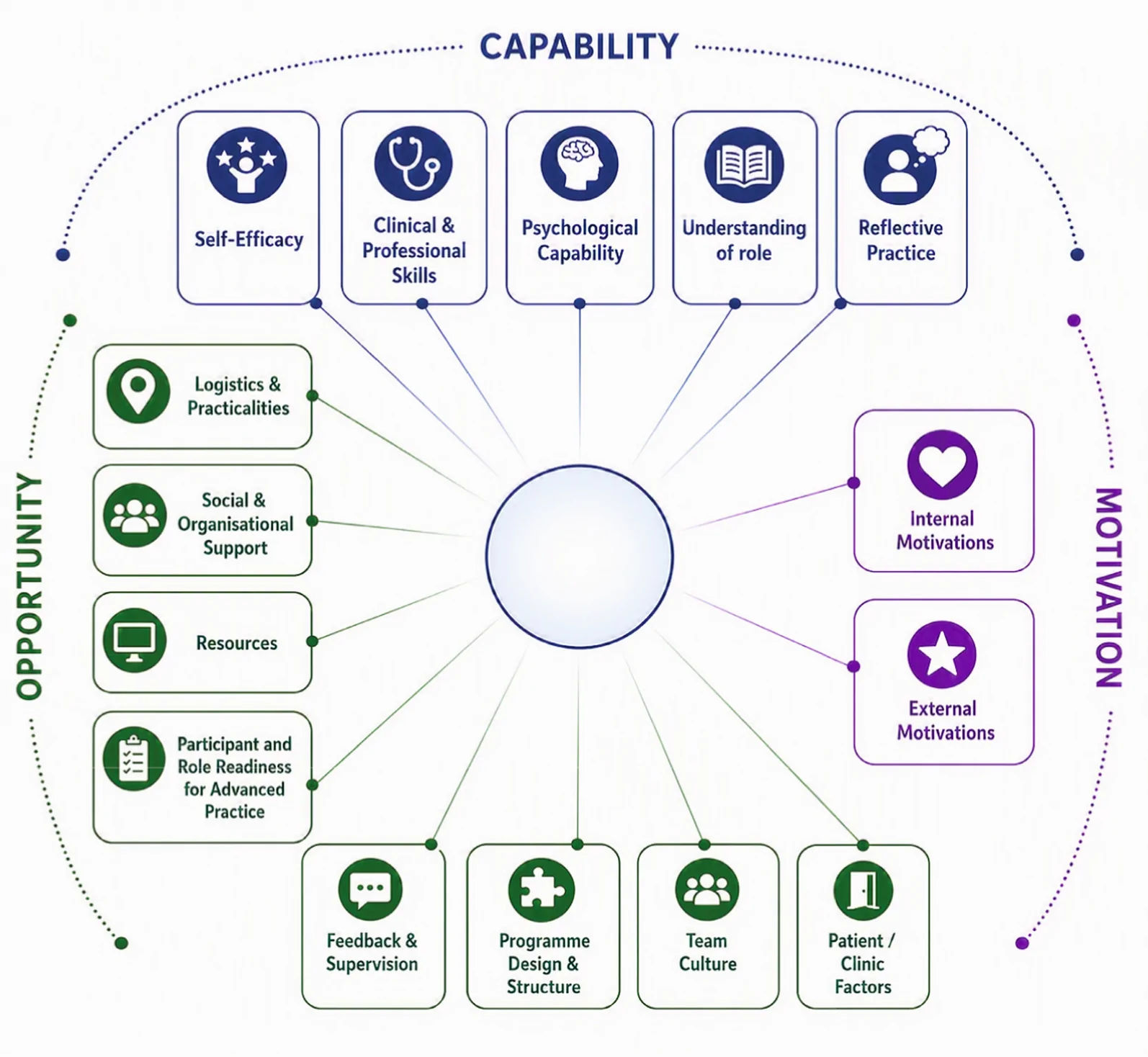
Participant perceptions of factors influencing implementation of a pilot preceptorship programme for pharmacists working towards the advanced career stage. Visual representation developed by the authors and refined using Microsoft Copilot (Microsoft Corporation) to support figure design

Participant perceptions of factors influencing implementation of a pilot preceptorship programme for pharmacists working towards the advanced career stage are shown in Fig. 2.

Stage 2 qualitative findings are presented below by COM-B domain.

### Capability

#### Preceptee capability

Capability was described across five themes (Fig. 2): self-efficacy, psychological capability (primarily resilience), clinical and professional skills aligned to RPS Core Advanced Curriculum, understanding of advanced practice (understanding of the knowledge, skills and behaviours required for advanced credentialling), and reflective practice. Capability was identified as a barrier for preceptees.

Low self-efficacy was frequently described in preceptees.

“I did wonder if my preceptee possibly didn't have the confidence… I don't think [they] had the confidence to do it.” Capability: Self-Efficacy

- Preceptor

Within psychological capability, resilience was identified as a barrier for preceptees, particularly in relation to managing workload pressures and seeking support.

“I think I probably could have benefited more from a bit more resilience and being a bit more vocal about more support that is needed” Capability: Psychological Capability

- Preceptee

Other perceived barriers included gaps in understanding of advanced practice, limited reflective capability, and clinical and professional skills, which were described as foundation level rather than working towards advanced practice.

“You know, clinical decision making has been more of an issue [for the preceptees]” Capability: Clinical and Professional Skills

- Senior Manager

“I would say [my preceptee’s clinical decision-making skills were at] foundation level which I never realised until we started this, but yeah, the more we got into it, I thought, oh, you know, [they’re] just not comfortable.” Capability: Clinical and Professional Skills

- Preceptor

#### Preceptor capability

For preceptors, capability was an enabler, examined across the same five capability themes.

“I don't have a whole like workload to block off that would then impact on resilience.” Capability: Psychological Capability

- Preceptor

#### Other stakeholders’ capability

For “other stakeholders”, capability was identified as a barrier. Self-efficacy, clinical and professional skills and understanding of advanced practice were viewed as barriers; psychological capability was neutral; and reflective practice was a seen as an enabler.

“I still think we're very risk averse and not in that grey space…and I know that there are a lot of our pharmacists out there that are not working to prescribing as they should” Capability: Clinical and Professional Skills

- Senior Manager

### Opportunity

#### Preceptee opportunity

Opportunity was viewed as a barrier for preceptees. Across the eight themes: logistics and practicalities, social and organisational support, resources, readiness for advanced practice (referring to suitability of participants’ baseline level and role-context fit for engaging in advanced practice), feedback and supervision, programme design/delivery (referring to clarity of expectations and implementation of the pilot programme), pharmacy team culture, and patient/clinic area factors, most themes were described as barriers, with only resources being viewed as an enabler.

Logistics and practicalities were thought to be a barrier for preceptees.

“The other work was always there, it was just always a case of oh, “I've, I've got too much, too much going on”, …more from [my preceptee’s] side.” Opportunity: Logistics and Practicalities

- Preceptor

Social and organisational support was also a perceived barrier for preceptees, with inconsistent stakeholder engagement limiting meaningful participation.

“Maybe like we could have had more support from like external stakeholders like GPs or the practices. I don't know if they were, maybe that aware of what was going on” Opportunity: Social & Organisational Support

- Preceptor

“I think what was challenging for us, or for me myself is because you're working in a private business. They have their own expectations of what work needs done, whereas it doesn't always line up from what you're trying to do” Opportunity: Social & Organisational Support

- Preceptee

Readiness for advanced practice was thought to be a barrier, with participants describing differences between expectations for advanced practice and substantive role requirements.

The theme of programme design and delivery was viewed as a barrier for preceptees, with participants describing a lack of clarity around expectations and support processes.

“[I would have benefitted from] clearer instruction on permission to ask for help early if struggling.” Opportunity: Programme Design & Delivery

— Preceptee

“Yeah, I think a clear description [at outset of programme] of what type of cases you want to see [would have assisted with full implementation].” Opportunity: Programme Design & Delivery

— Preceptor

Culture within the pharmacy team was described as a barrier for preceptees, with participants describing a lack of development in the preceptee peer group.

“I do feel there is a whole cohort almost who have missed out on a lot of the structured training and my preceptee's probably in that camp” Opportunity: Pharmacy Team Culture

— Preceptor

Feedback and supervision was perceived to be a barrier, with participants describing limited feedback, particularly in relation to practical aspects of clinic delivery and organisation.

Resources, including learning materials and clinical equipment, were described as an enabler for preceptees.

#### Preceptor opportunity

For preceptors, the Opportunity domain was predominantly viewed as an enabler. Four themes were enablers (logistics and practicalities, social and organisational support, resources, and readiness for advanced practice), two were barriers (feedback and supervision; programme design/delivery), and two were not reported (pharmacy team culture; patient/clinic area factors).

Enablers described reflected established self-efficacy, resilience, protected clinical time, stakeholder understanding of and autonomy within role, and access to resources. Feedback & Supervision was thought to be a barrier due to missed opportunities for preceptors to provide feedback to preceptees on the operational aspects of clinic delivery and set-up. Programme Design/Delivery was also viewed as a barrier, with transcripts suggesting some lack of clarity around induction and expectations at the outset, reflected in variability in consistency of feedback on non-clinical skills.

“So, I had three days a week where I had clinics, so there were no issues in terms of clinic room space.” Opportunity: Logistics and Practicalities

- Preceptor

#### Other stakeholders’ opportunity

For other stakeholders, the Opportunity domain was perceived as a barrier, with variation across themes. Social and organisational support, pharmacy team culture, logistics and practicalities, and feedback and supervision were viewed as barriers, with issues reported around engagement with the programme and clarity of roles in implementation. Resources and readiness acted as enablers, while programme design/delivery showed a mixed pattern, with both enabling and limiting elements described across transcripts. Patient/clinic area factors were not reported for this group.

### Motivation

#### Preceptee motivation

Motivation for preceptees was mixed overall. The domain comprised two themes: internal and external motivation. Internal motivation to achieve advanced practice was generally present and related to the drive to develop confidence, clinical knowledge and help others as well as the perceived value of preceptorship. However, accounts also suggested that confidence-related factors at times constrained motivation, with some participants questioning whether preceptee motivation was sustained throughout the programme. In contrast, external motivation was described as a barrier, with limited reinforcement from formal recognition or portfolio-related incentives insufficient to sustain engagement. Overall, motivation was therefore interpreted as mixed due to the balance between enabling internal drivers and limiting external factors.

“Once we were into it, I wasn't convinced that [my preceptee] did [have the motivation]. I don't know whether that was just a bit of fear in [my preceptee]. I wasn't sure about my preceptee. If [they] really did want to be involved. Motivation: Internal.

- Preceptor

“I think [the preceptorship programme gave me] an improved…more confident clinical knowledge, a new like relationship like with another pharmacist” Motivation: Internal.

- Preceptee

#### Preceptor motivation

For preceptors, motivation was described as an enabler. Both internal and external motivation supported engagement with the programme. Internal motivation included a commitment to supporting others, advancing the profession, and fostering a culture of learning and improvement. External motivation included drivers such as advanced portfolio completion and engagement with the preceptor role.

#### Other stakeholders’ motivation

For other stakeholders, particularly senior managers, motivation acted as an enabler overall based on available data. Internal motivation focused on supporting teams in delivering and developing advanced practice. External motivators were not described for this group.

## Discussion

### Summary of key findings

In this pilot preceptorship programme for pharmacists working towards advanced practice, implementation was incomplete, with fewer joint clinical sessions and review meetings delivered than planned. Despite this, preceptees who completed the programme reported some changes in self-efficacy and competence across selected RPS Core Advanced Curriculum outcomes. Analysis using the COM-B framework suggested that constraints in opportunity including logistics, protected time and stakeholder engagement were thought to be the main barriers to programme delivery, while capability was thought to be an additional barrier. Qualitative findings relating to capability could be interpreted as reflecting a potential mismatch between preceptee experience, organisational scaffolding, and the requirements of structured advanced-level supervised practice, suggesting a need for more explicitly staged developmental support. Motivation among participants was generally supportive, contributing positively where opportunity and capability allowed engagement.

### Strengths and weaknesses

#### Strengths

A key strength of this study is its mixed-methods design, combining quantitative measures of self-efficacy and competence with qualitative exploration of perceived barriers and enablers, providing both measurable outcomes and contextual insight. The qualitative analysis is rigorous, using the COM-B framework to systematically examine factors influencing preceptorship implementation in advanced pharmacist practice. Alignment with the RPS Core Advanced Curriculum ensures the findings are relevant to current professional standards, and the study offers early, real-world evidence on feasibility findings within a small-scale pilot.

#### Weaknesses

This study was a small-scale pilot, with only three pairs completing the programme, limiting generalisability. Quantitative outcomes were self-reported, which may introduce response bias, and the competence measure was adapted from previous work but not formally validated. The study was conducted within a single NHS board and focused on primary care, so findings may not transfer to other organisational or sectoral contexts. Qualitative analysis was based on a small, fixed sample of participants involved in a single pilot programme. Findings represent subjective participant accounts and should therefore be interpreted as perceptions of factors influencing implementation rather than definitive explanations for programme outcomes. The limited sample restricted the breadth of perspectives captured and may not have fully represented the range of factors influencing implementation. Only participants who completed Stage 1 were invited to qualitative interviews, which may have limited insight into barriers experienced by early non-completers. Participants were identified through managerial nomination, which may have introduced selection bias; however, the direction or magnitude of any such effect is unknown.

#### Interpretation

Preceptorship programmes, as a form of evidence-based clinical supervision [15–18], are intended to support the development of self-efficacy and competence in preceptees. However, implementation was incomplete, highlighting challenges in feasibility and a broader disconnect between intervention design and organisational readiness in practice [28]. In this study, limited joint clinical sessions, incomplete review meetings, and variable engagement illustrate that preceptees may be operating in a system not fully prepared to support advanced practice. This finding aligns with wider UK workforce analyses, which highlight a persistent gap between national ambitions for pharmacist prescribing and the organisational conditions required to support implementation in practice [29].

Similar implementation challenges, including perceived barriers related to capability (confidence and clinical skills), opportunity (logistics, protected time, and stakeholder support), and motivation, have been reported in other pharmacy workforce development initiatives [30–33] This study extends existing research by identifying views on where constraints become most apparent in practice. These findings indicate that the model of structured clinical supervision implemented in this pilot, intended to support development of advanced pharmacists in line with workforce transformation goals [2, 8], was not feasible within this context. This highlights how pilot programmes can inform future workforce development by exposing gaps between intended intervention design and the realities of practice.

Robust evaluation of advanced pharmacist preceptorship programmes under full implementation is required to understand the full potential of this support. Using the COM-B framework provided insight into participants’ perceptions of factors contributing to incomplete implementation of the pilot programme. Participants identified opportunity constraints, including clinic logistics, lack of protected time, and low stakeholder engagement, and capability factors, such as underdeveloped clinical decision-making skills and low resilience, should be understood and addressed in future work in this area. Motivation was generally thought to be an enabler, shaping successful engagement where opportunity and capability constraints allowed.

These findings align with previous pharmacy workforce studies showing that expectations for pharmacist roles are often not matched by organisational support [28–32]. Reported gaps include limited protected time, inconsistent supervision and mentorship, variable managerial engagement, and insufficient resources and infrastructure to enable safe practice. This finding reinforces recommendations that pharmacists working towards advanced practice must be supported by organisations providing clear guidance, structured support, and developmental resources to progress safely and confidently [9], and further suggests that while individual pharmacists are expected to meet evolving professional standards, such expectations cannot be reliably realised without appropriate organisational conditions, with responsibility for enabling advanced practice therefore primarily located at system level. Overall, the findings suggest that advanced-level preceptorship is unlikely to be effective without corresponding organisational conditions that actively enable, resource, and normalise supervised clinical development within routine practice.

### Further research

Further research into the effect of pharmacist preceptorship programmes to support advanced practice is required, using insights from this pilot to guide study design and evaluation. Exploring how participant capability, opportunity, and motivation interact with organisational context, and how these factors influence programme outcomes, would be valuable. More broadly, the role of strengthened clinical supervision, of which preceptorship is one strand, should be examined to understand how it can better support pharmacists’ development.

## Conclusion

This pilot preceptorship programme for pharmacists working towards advanced practice was not feasible, with fewer joint clinical sessions and review meetings delivered than planned. Despite incomplete implementation, participants who completed the programme reported changes in self-efficacy and competence across some RPS Core Advanced Curriculum outcomes. Analysis using the COM-B framework highlighted that views on constraints in opportunity, including logistics, protected time, and stakeholder engagement, were the main perceived barriers, while capability was viewed as posing additional challenges. Motivation was generally seen as an enabler. These findings suggest that targeted strategies addressing organisational and contextual barriers are needed to optimise implementation of preceptorship programmes and support pharmacists working towards advanced practice.

## Supplementary Information

Below is the link to the electronic supplementary material.

Supplementary file1 (DOCX 23 kb)

## Data Availability

The participants of this study did not give written consent for their data to be shared publicly, so due to the sensitive nature of the research, supporting data is not available.
