# Supplementary material for: Feasibility of a pilot preceptorship programme for pharmacists working towards the advanced career stage in Scotland: a mixed-methods study
Source: Int J Clin Pharm. 2026 Sep 18;48(6):2456–66. doi: 10.1007/s11096-026-02222-z (PMC13731518; doi:10.1007/s11096-026-02222-z)
Supplement: Supplementary file 1 [file 11096_2026_2222_MOESM1_ESM.docx]

**Supplementary Files**

Supplementary Files 1–3: (1) Modification of instruments, (2) COM-B topic guide, (3) Coding framework and codebook **1 - Modification of self-efficacy and competence instruments for advanced pharmacist practice**

**Development of pilot outcome measures**

Preceptee self-reported self-efficacy and competence measures used in this pilot study were derived from existing published instruments and underwent minor contextual modifications to reflect advanced pharmacist practice and Domain 2 (“Professional Practice”) outcomes of the Core Advanced Pharmacist Curriculum.

**Self-efficacy (situational confidence)**

Self-efficacy items were based on a validated General Self-Efficacy Scale hosted by Imperial College London [24]. The original scale measures situational confidence using a 5-point Likert response structure.

Original item format:
Participants are asked:

“How confident do you feel about performing the following tasks?”

Participants then rate task-specific items using the response scale below.

Original response structure:

1. Not confident at all
2. Not very confident
3. Neutral
4. Quite confident
5. Extremely confident

For this pilot study, only minor wording modifications were made to contextualise items to advanced pharmacist practice and Domain 2 curriculum outcomes (2.1–2.3). The ordinal response structure and underlying construct of self-efficacy (situational confidence) were retained, with no changes to scale format or scoring structure.

Modified item stem used in this study:

“How confident did you feel to deliver the following Domain 2 (Professional Practice) outcomes?”

**Competence (self-reported competence)**

Competence items were based on the self-perceived competence scale described by Lai et al. (2007) [25], which has been widely used in published studies.

The original scale uses a 5-point progression from lack of competence through to independent practice. For this pilot study, minor contextual wording modifications were made to align descriptors with advanced pharmacist clinical practice and supervision structures. The progressive competence construct and 5-point ordinal structure were retained.

**Original item format:**
Participants are presented with a competence progression scale and asked to rate their level of perceived competence for each task or outcome.

**Original response structure:**

1. Grossly inadequate
2. Know the approach in theory, not confident at all in real situations
3. Only confident in making certain decisions, need seniors to be readily available or on constant standby
4. Reasonably confident, but need seniors who are contactable for consultation
5. Very confident, can be relied on without supervision

For this pilot study, the descriptors were modified to reflect advanced pharmacist practice roles, clinical decision-making responsibilities, and supervision arrangements, while retaining the original 5-point ordinal structure and underlying construct of self-perceived competence.

**Modified descriptors used in this study:**

1. I am not at all competent
2. I am competent in the theory but not in delivery in practice
3. I am competent to deliver this outcome for certain patients but would usually prefer to discuss decisions with seniors
4. I am competent to deliver this outcome for most patients, only occasionally requiring discussion with seniors
5. I am fully competent and can be relied upon to deliver this outcome

**Mapping to Advanced Pharmacist Curriculum outcomes**

Both instruments were mapped directly to Domain 2 (“Professional Practice”) outcomes 2.1–2.3 of the Core Advanced Pharmacist Curriculum. No changes were made to:

- the number of response categories,
- ordinal scaling direction,
- progression of competence/confidence constructs,
- or frequency of measurement.

Only minor contextual wording changes were introduced to improve relevance to advanced pharmacist clinical practice.

**2 – COM-B Interview Topic Guide**

Semi-structured interview guide informed by the COM-B model, developed to explore implementation of a pilot preceptorship programme for pharmacists working towards the advanced career stage.

The interviewer will introduce themselves and explain the interview format. The following questions and prompts will be used in semi-structured one-to-one interviews.

**Capability:**

1 - Do those involved* in the pilot** have the capability (required skills) to engage fully and deliver their role?

Prompts

- Do those involved* have the required confidence?
- Do those involved* have the required mental skills?
- Do those involved* have the required memory?
- Do those involved* have the required resilience?
- Do those involved* have the right tools?
- Do those involved* have the required equipment?
- Do those involved* have the required physical capability?
- Prompt to consider *all* of those involved*

**Opportunity:**

2 - Do those involved* in the pilot** have the opportunity (required circumstances) to engage fully and deliver their role?

Prompts

- Is the pilot deliverable in their physical environment?
- Do those involved* have enough time to engage fully with the pilot?
- Do those involved* have enough support from others to engage fully with the pilot?
- Do those involved* have the right resources, like equipment (including preceptorship materials) to engage fully with the pilot?
- Prompt to consider *all* of those involved*

**Motivation:**

3 - Do those involved* in the pilot** have the motivation to engage fully with the pilot?

Prompts

- What are the main reasons those involved* wanted to be part of the pilot?
- What are the benefits of the pilot?
- What were your concerns or worries about taking part in the pilot?

Prompt to consider *all* of those involved*

*Those involved refers to all pilot participants (preceptees, preceptors and supporting senior management). When answering, the participant may choose to talk about themselves, others or both and the interviewer may prompt them to consider all involved.

**Pilot refers to pilot preceptorship programme for pharmacists working towards the advanced career stage.

**3 - Coding Framework and Codebook**

| Deductive COM-B Domain | Inductive COM-B themes | Code | Inductive COM-B Code |
| --- | --- | --- | --- |
| Capability | Self-Efficacy (confidence in advanced practice) | C1.1 | Confidence in Advanced Practice Delivery |
|  |  | C1.2 | Comfort, familiarity and ease of interaction with portfolio completion |
|  | Clinical & Professional Skills | C1.3 | Clinical Decision-Making Skills |
|  |  | C1.4 | Strong Consultation Skills |
|  |  | C1.5 | Ability to Apply Knowledge in Practice |
|  | Psychological Capability | C1.6 | Participants had Required Resilience |
|  | Understanding of Advanced Practice | C1.7 | Understanding of the Core RPS Curriculum |
|  | Reflective Practice | C1.8 | Ability to Reflect on Practice and Adapt |
| Opportunity | Logistics & Practicalities | O2.1 | Base (Geography) / Working Days Logistically Appropriate |
|  |  | O2.2 | Clinics Logistically Appropriate (space, set up, arrangement, admin support) |
|  |  | O2.3 | Protected Time |
|  |  | O2.4 | Ease of completion of programme activities (including clinics, objective setting, review meetings, evaluation) |
|  | Social & Organisational Support | O2.5 | Stakeholder Engagement |
|  |  | O2.6 | Integration into Practice Team |
|  |  | O2.7 | Organisational Support |
|  |  | O2.8 | Strong peer support/professional networks |
|  |  | O2.9 | Pharmacy team appropriately skill mixed to support advanced practice |
|  |  | O2.10 | Strategic Leadership Support for Advanced pharmacist practice |
|  | Resources | O2.11 | Access to resources (learning/clinical/equipment) |
|  | Readiness for advanced practice | O2.12 | Participant(s) experienced and working at correct level |
|  |  | O2.13 | Role of preceptee ready for advanced practice |
|  | Feedback & Supervision | O2.14 | High quality feedback/supervision |
|  | Programme Design /Delivery | O2.15 | Strong, appropriate inclusion criteria for programme (programme design) |
|  |  | O2.16 | Inclusion criteria for programme was accurately adhered to (actual selection) |
|  |  | O2.17 | Clear Programme Structure and induction |
|  | Pharmacy Team Culture | O2.18 | Workplace culture of advanced pharmacist practice in action |
|  | Patient/Clinic area Factors | O2.19 | Patient engagement |
|  | Internal Motivations | M3.1 | Motivation to Achieve Advanced Practice Level |
|  |  | M3.2 | Perceived Value of Preceptorship |
|  |  | M3.3 | Growth Mindset |
|  |  | M3.4 | Motivation to improve patient care/outcomes |
|  |  | M3.5 | Motivation to help peers/colleagues/MDT |
|  |  | M3.6 | Motivation to engage in advanced practice is increased by feeling valued or recognised. |
|  | External Motivations | M3.7 | Completion of the Core Advanced Portfolio drives motivation |
|  |  | M3.8 | Completion of the Preceptorship Programme drives motivation |
|  |  | M3.9 | Career progression/promotion drives motivation |
